# Effect of Build Orientation and Part Location on Surface Roughness of PA12 Components Fabricated by Selective Laser Sintering

**DOI:** 10.3390/polym18121415

**Published:** 2026-06-06

**Authors:** Lokeshwaran Srinivasan, Ezhilmaran Veeranan, Lalitha Radhakrishnan, Monaf Hodhod, Skander Jribi, Mohammad Faseeulla Khan

**Affiliations:** 1 Department of Manufacturing Engineering, Anna University, Chennai 600025, India; 2 Department of Mechanical Engineering, College of Engineering, King Faisal University, Hofuf 31982, Al-Ahsa, Saudi Arabia

**Keywords:** laser powder bed fusion, Polyamide 12, surface roughness, porosity, crystallinity index

## Abstract

Selective laser sintering (SLS) enables the fabrication of polymer components with intricate geometries; however, surface roughness remains a concern that affects performance and quality. This study systematically examines the influence of build orientation (X, Y, Z) and part location (inner, middle, outer) on surface roughness of Polyamide 12 (PA12) components. The results show that components built in the Y orientation and placed in the inner chamber exhibited the lowest surface roughness (Sa = 9.88 ± 0.16 µm). In contrast, Z-oriented components at the outer edge showed the highest (Sa = 11.49 ± 0.27 µm), indicating a 10.13% variation. SEM analysis showed smoother morphology and reduced porosity in the inner regions, while DSC confirmed higher crystallinity in samples with improved finish. These findings highlight the role of thermal stability and spatial positioning within the build chamber in controlling surface characteristics, emphasizing the importance of orientation and placement strategies for improving the quality of PA12 components fabricated by SLS.

## 1. Introduction

Selective laser sintering (SLS) is a widely adopted laser powder bed fusion (LPBF) additive manufacturing (AM) technique that allows the fabrication of polymer components with high accuracy [1,2,3]. This layer-by-layer fabrication method, commonly using PA12 powder, removes the requirement of inherent support structures and allows high packing density of parts within the build chamber [4,5]. However, despite its advantages, parts made using SLS often have a rougher surface finish than those made through traditional methods, such as injection molding [6,7]. During SLS, surface roughness arises due to several mechanisms, including incomplete melting of powder particles, particle agglomeration, stair-stepping effects from layer-by-layer construction, and powder adhesion to partially molten surfaces [8,9].

Surface roughness is a critical quality parameter in SLS-fabricated polymer components because it directly influences dimensional fitting, wear resistance, fatigue behavior, and interfacial interactions in functional applications. In aerospace and automotive applications, excessive surface roughness can increase aerodynamic drag, reduce fatigue strength, and affect the assembly accuracy of lightweight structural components. Similarly, in mechanical assemblies, rough surfaces may increase friction and wear during repeated contact operations, thereby reducing component lifespan. Furthermore, poor surface finish often necessitates additional post-processing treatments, which increase manufacturing cost and production time. Therefore, controlling surface quality during fabrication is essential for improving the functional performance, dimensional reliability, and service life of SLS-fabricated PA12 components [10,11,12,13]. Several studies have investigated the influence of laser processing parameters, namely laser scan speed, layer thickness, laser power, and hatch distance, on the surface roughness of parts fabricated through SLS [14]. Advances in measurement techniques have further improved the ability to analyze surface texture variations in SLS-printed parts [15].

Researchers have explored various methods for characterizing and quantifying surface roughness in SLS parts. Launhardt et al. compared various measurement techniques, including profilometry, focus variation, and confocal laser scanning microscopy [16]. Petzold et al. investigated the effects of laser power, laser scan speed, and powder characteristics, demonstrating that optimizing these parameters is essential for minimizing surface irregularities [17]. Tonello et al. studied the relation between the grain size of the powder and surface roughness in polyamide-based SLS parts, highlighting the influence of powder characteristics and laser exposure settings on surface morphology [18]. Additionally, process parameters such as temperature and cooling rates can affect material crystallinity, with rapid cooling often leading to lower crystallinity and a rougher surface finish. Therefore, optimizing the build parameters is necessary to understand the optimal relationship between crystallinity and surface quality.

The literature review Indicates that higher laser power can cause excessive melting, altering the surface texture, while lower power can result in incomplete fusion, leading to rougher surfaces. Scan speed and hatch spacing are also crucial, as improper settings can lead to uneven heat distribution, which in turn contributes to surface roughness [19,20]. Moreover, powder properties such as powder particle size and flowability affect layer deposition uniformity, which directly influences surface finish [10].

Build orientation is one of the key factors that affects the surface properties of components fabricated using SLS. The orientation of a part during printing determines the stacking of layers, directly impacting surface quality. It is primarily due to the staircase effect and inconsistencies in laser exposure across different orientations [21]. Similarly, the placement of components within the build chamber plays a crucial role, as thermal gradients and inconsistencies in powder recoating can lead to variations in surface quality [22,23]. Recent studies have reported that build orientation significantly affects surface morphology due to the staircase effect, layer formation, and partially fused powder adhesion during fabrication. Additionally, variations in thermal distribution and cooling behavior within the build chamber can influence fusion quality and dimensional stability of fabricated parts [24,25]. Understanding these influences is essential for optimizing SLS processes to achieve better surface finishes and dimensional accuracy while minimizing the need for post-processing. This study aims to provide a deeper understanding of these factors, offering insights that can guide improved building strategies for enhanced manufacturing outcomes.

While previous studies have primarily focused on dimensional accuracy, investigating deviations caused by thermal stresses, warping, and shrinkage, fewer have explored the influence of build parameters, such as part location and orientation, on surface roughness [26]. A more detailed investigation is necessary, as part placement within the build chamber affects heat distribution, cooling rates, and the adhesion of unsintered powder. Although existing research has established correlations between build parameters and dimensional deviations, this study focuses on understanding their impact on surface roughness, a critical factor for functional applications and post-processing requirements.

Although previous studies have investigated the influence of process parameters such as laser power, scan speed, and hatch spacing on surface roughness and dimensional accuracy of SLS-fabricated PA12 components, limited attention has been given to the combined influence of build orientation and spatial part location within the build chamber. In particular, the relationship between thermal gradients, crystallinity variation, porosity evolution, and surface morphology across different chamber zones remains insufficiently explored. Therefore, the present study systematically investigates the coupled effects of build orientation and part location on the surface roughness, dimensional Deviation, SEM morphology, and DSC-based crystallinity behavior of PA12 components fabricated using selective laser sintering.

## 2. Materials and Methods

### 2.1. Process Parameters Selection

**Figure 1:**
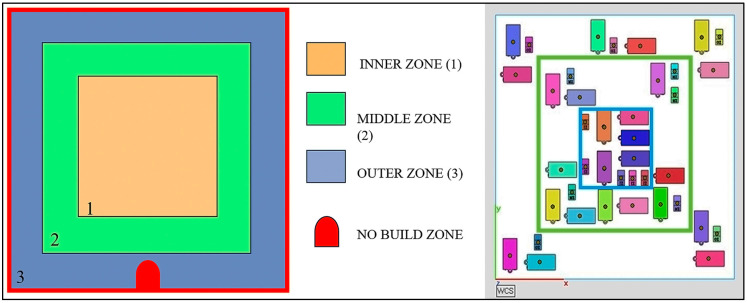
Schematic representation of different zones in the build platform.

Based on earlier studies, build orientation and part location were considered as key parameters influencing the surface roughness of SLS components. As adapted from prior research, an experimental study was conducted by fabricating samples in different positions and orientations [25]. During the cooling phase, heat dissipates more rapidly in the peripheral regions of the build chamber than in the inner areas, which affects surface roughness. In earlier research, Allison et al. [26] classified part locations into two major regions. However, based on findings by Rupalin et al., the part location is further divided into three zones, as shown in Figure 1. In this classification, the outer zone experiences the fastest heat dissipation, the middle zone has a moderate dissipation rate, and the inner zone retains heat for the longest duration.

### 2.2. Fabrication of the Samples Through Selective Laser Sintering

**Figure 2:**
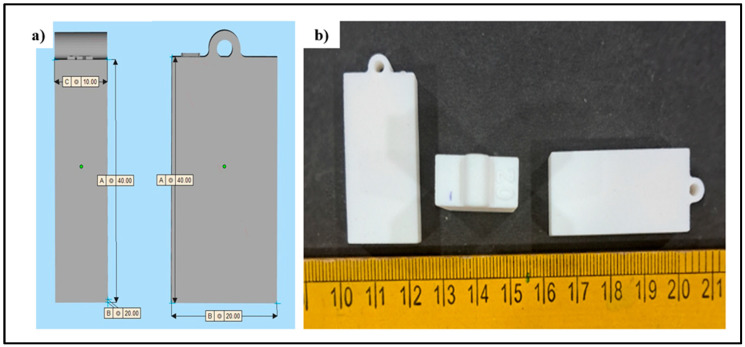
(**a**) Designed component and (**b**) fabricated cuboid component.

In this study, a total of 45 cuboid test components of dimensions (40 × 20 × 10 mm^3^) for 9 experimental runs based on the Taguchi L9 orthogonal array. For each run, five samples were fabricated to ensure repeatability, as shown in Figure 2. PA12 powder supplied by EOS GmbH was used in this study. The powder exhibited a particle size distribution ranging from 20 to 80 µm with an average particle diameter of 56 µm. The semi-crystalline polymer powder possessed good flowability and near-spherical morphology, which facilitated uniform powder spreading during fabrication. The powder consisted of 50% virgin powder and 50% recycled powder collected from previous build cycles. The recycled powder had undergone no more than 3 reuse cycles to minimize thermal degradation effects. Prior to fabrication, the powder was dried at 25 ± 2 °C for 24 h to remove absorbed moisture and ensure stable powder flowability during the SLS process. The components were fabricated using a Selective Laser Sintering (SLS) (EOS P396, EOS GmbH, Krailling, Germany) system equipped with a CO_2_ laser source. The fabrication process was carried out using a laser power of 40 W, scan speed of 2500 mm/s, hatch spacing of 0.3 mm, and layer thickness of 120 µm. During processing, the build chamber temperature was maintained at 172 °C, while the removal chamber temperature was set at 130 °C to ensure stable thermal conditions for PA12 powder sintering. After completing each layer, the build platform was lowered by the thickness of one layer, and the cycle was repeated. Once printing was finished, the components were left to cool before being removed from the powder bed. The excess powder was post-processed using shot blasting.

### 2.3. Measurement and Characterization

Surface roughness measurements were performed using a non-contact surface roughness tester (Taylor Hobson, AMETEK, Leicester, England). The surface morphology and areal roughness characteristics of the fabricated PA12 components were evaluated at a magnification of 10× with a measuring area of 1.65 × 1.65 mm. To ensure reliable characterization and minimize the influence of localized surface irregularities, measurements were obtained from three different locations distributed across the specimen surface. The areal surface roughness parameters were evaluated according to ISO 25178 standards [27]. Arithmetic mean height (Sa) represents the average Deviation of surface peaks and valleys from the mean plane. Root mean square roughness (Sq) provides the standard Deviation of surface height distribution and is more sensitive to larger deviations. Maximum surface height (Sz) indicates the total height difference between the highest peak and the lowest valley within the measured area. Standard deviation analysis was additionally carried out to assess the repeatability and consistency of the experimental measurements.

Dimensional accuracy of the fabricated components was evaluated using digital vernier callipers with a resolution of 10 µm. Measurements were conducted along the X, Y, and Z directions, and each dimension was measured five times to reduce experimental uncertainty. The average measured value was used to determine the dimensional Deviation (DD) of the fabricated components. The dimensional Deviation was calculated as the difference between the measured dimension and the nominal CAD dimension of the specimen.

The surface morphology and porosity characteristics of the fabricated samples were further examined using a scanning electron microscope (SEM) (VEGA3, TESCAN, Bron, Czech Republic). SEM analysis was carried out to investigate the fusion behavior, pore formation, and surface texture variations associated with different build locations within the SLS chamber.

Differential scanning calorimetry (DSC) analysis was performed using a NETZSCH DSC instrument (NETZSCH, Selb, Germany) to evaluate the crystallinity behavior of the fabricated PA12 samples. The DSC equipment had a resolution of 1 µW and a temperature range up to 1400 °C. The analysis was conducted to examine the influence of thermal conditions and build location on the crystallization behavior of the SLS-fabricated components.

## 3. Results and Discussion

Surface roughness evaluation was carried out based on a Design of Experiments (DoE) framework using a Taguchi L9 orthogonal array, as presented in Table 1. This approach enabled a systematic investigation of the effect of key process parameters, specifically build orientation and part location, while minimizing the number of experimental trials required. Statistical analysis was performed using the Minitab 17 statistical software, where Analysis of Variance (ANOVA) [28] was used. These analyses quantified the individual contributions of build orientation and part location to surface roughness.

### 3.1. Effect of Variables on Surface Roughness

**Figure 3:**
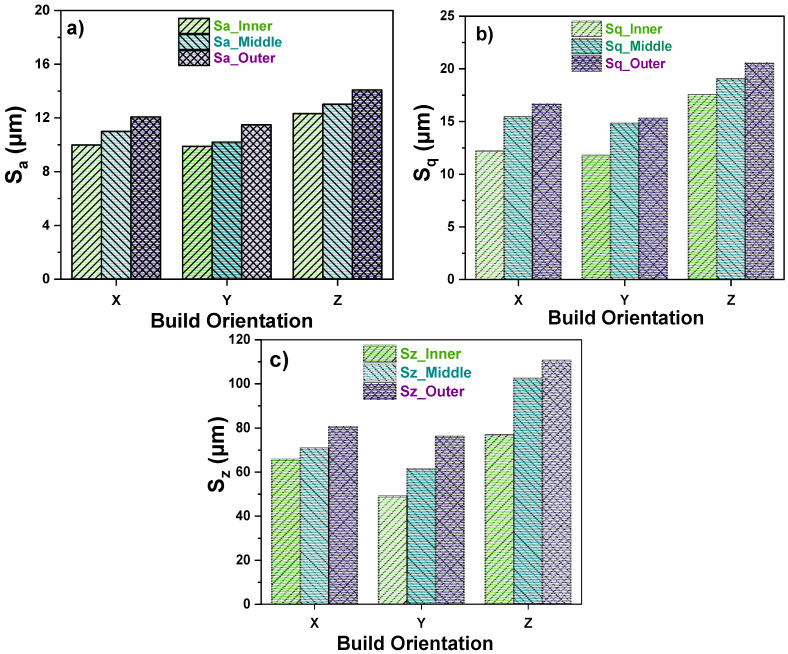
Effect of build orientation on surface parameters (**a**) Sa, (**b**) Sq, (**c**) Sz.

Build orientation has a significant impact on surface roughness due to the layer-wise nature of the SLS process. From Table 1, it has been observed that components oriented in the X-direction have S_a_ values ranging from 9.98 ± 0.18 µm to 12.07 ± 0.29 µm. In contrast, those in the Y-orientation vary from 9.88 ± 0.16 µm to 11.49 ± 0.27 µm. The Z-orientation exhibited the highest roughness, with Sa reaching 14.08 ± 0.41 µm. Similar trends were observed for S_q_ and S_z_, as shown in Figure 3, where Z-oriented components showed maximum roughness of 20.59 ± 0.71 µm and 110.54 ± 3.48 µm, respectively. This elevated roughness is primarily attributed to the stair-stepping phenomenon, which becomes more pronounced when vertical features are built perpendicular to the layer plane.

**Figure 4:**
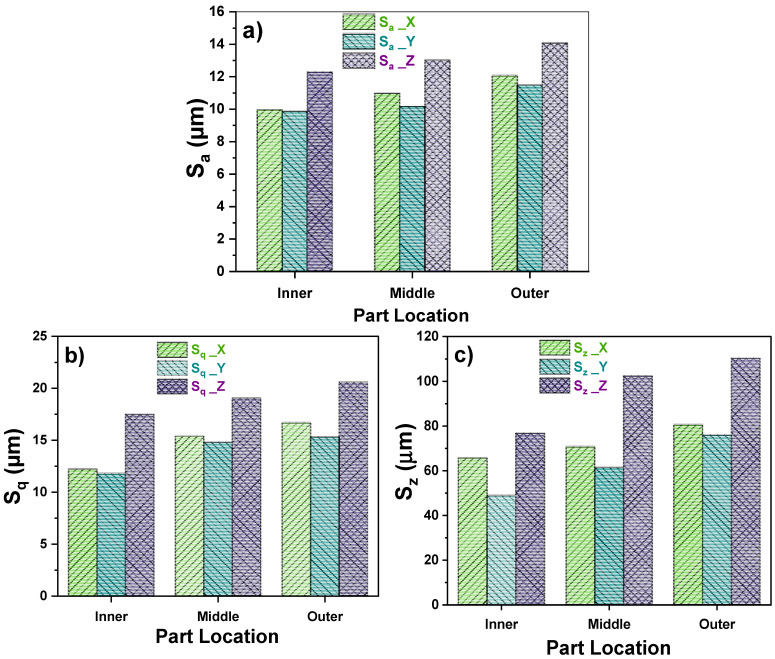
Effect of part location on surface parameters (**a**) Sa, (**b**) Sq, (**c**) Sz.

In contrast, X and Y-oriented components undergo more uniform laser scanning, which promotes smoother surfaces through improved interlayer bonding, resulting in reduced surface roughness [29]. Moreover, the location of components within the build chamber also has a crucial role in surface roughness due to differences in heating and cooling rates. Components located in the inner region of the platform experienced lower S_a_ values (as low as 9.88 µm) (Figure 4), likely due to more stable and uniform heat retention in the central region. PA12’s low thermal conductivity allows the powder bed to retain heat longer in the center, facilitating consistent fusion and smoother surface formation [30]. Components located in the middle regions of the build chamber show intermediate surface roughness values, reflecting the influence of moderately varying thermal gradients and transitional cooling behavior. These conditions result in a surface quality that lies between those observed in the inner and outer zones. Conversely, components situated near the outer edges experienced higher S_a_ values, reaching 14.08 µm, along with increased S_q_ and S_z_. It is due to more rapid heat dissipation at the chamber boundaries, where exposure to cooler surrounding air accelerates solidification [31]. These faster cooling rates can hinder proper layer fusion, resulting in greater surface irregularities.

A comparison of the two factors reveals that build orientation has a stronger influence on all roughness parameters than part location. However, the contribution of part location is also notable. Surface roughness increased by approximately 8.5–18% when transitioning from inner to outer part locations, and even more substantially when changing from X- or Y-orientation to Z-orientation. As shown in Table 2, the ANOVA results indicate that both build orientation and part location significantly influence the surface roughness parameters, as all *p*-values were <0.05. Central regions support more consistent sintering, leading to smoother surfaces. The predictive models show excellent accuracy, as indicated by their high goodness-of-fit values, confirming their effectiveness in guiding surface quality improvements in SLS components.

**Figure 5:**
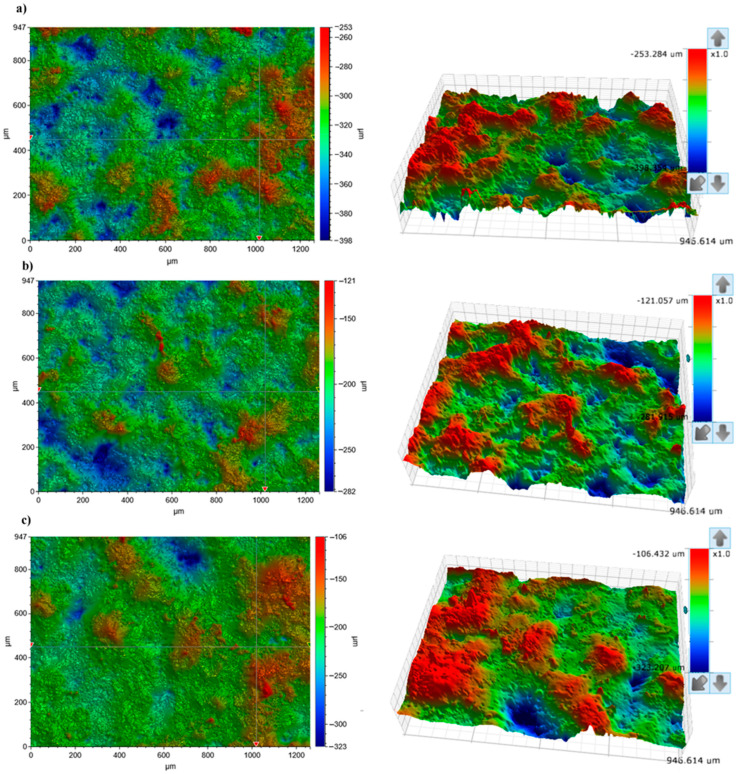
Non-contact surface roughness 3D graph for inner position component: (**a**) X direction, (**b**) Y direction, and (**c**) Z direction.

The combined effect of build orientation and part location reveals that the lowest surface roughness values are observed in components fabricated in the Y-orientation and positioned in the inner regions of the build chamber. The non-contact surface roughness results of the components placed in the inner region are shown in Figure 5. These results highlight the combined effects of orientation and part placement, emphasizing the need for strategic part arrangement within the SLS build chamber to minimize surface roughness and improve the consistency of printed components.

### 3.2. Effect of Variables on Dimensional Deviation

**Figure 6:**
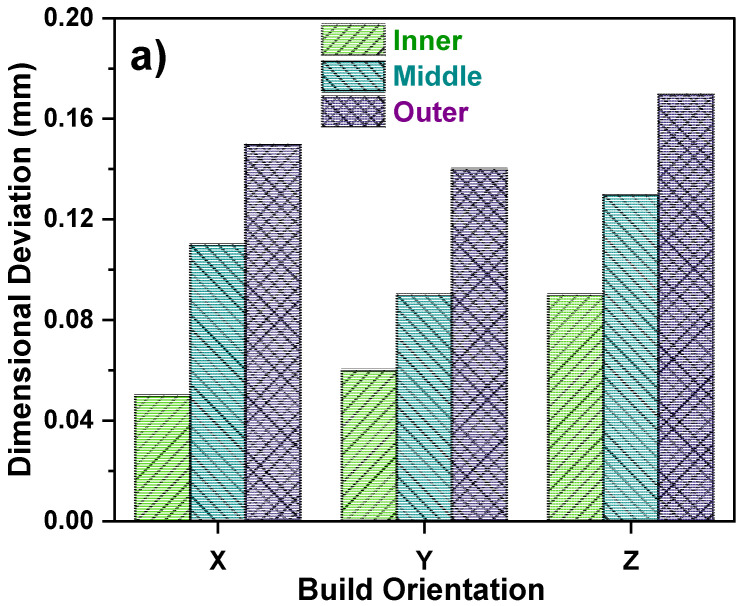

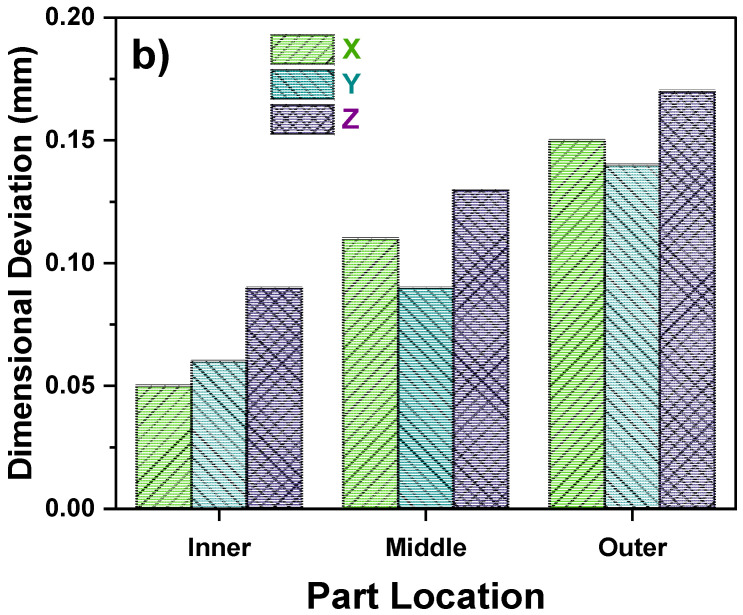
(**a**) Effect of build orientation vs. dimensional Deviation and (**b**) Part location vs. dimensional Deviation.

Dimensional Deviation in SLS-fabricated components is significantly influenced by build orientation and part placement within the build chamber. As shown in Figure 6a, components built along the *Y*-axis exhibited the least Deviation (0.05 ± 0.003 mm to 0.15 ± 0.008 mm). Although SLS employs an alternative hatching strategy to mitigate laser scan direction effects, minor variations persist, particularly in X-oriented components (0.06 ± 0.004 to 0.14 ± 0.007 mm) [32]. Z-oriented components showed the highest deviations (0.09 ± 0.005 mm to 0.17 ± 0.009 mm), likely due to cumulative errors from vertical stacking and increased shrinkage [33]. The increased dimensional Deviation in the Z-orientation can be attributed to the cumulative effect of layer stacking and thermal shrinkage occurring along the build direction. Since the *Z*-axis is perpendicular to the powder bed, repeated thermal cycling and layer-by-layer solidification increase residual stresses and non-uniform shrinkage, thereby reducing dimensional accuracy. In contrast, the X- and Y-oriented components experienced relatively uniform heat distribution and reduced thermal distortion, resulting in improved dimensional consistency.

Figure 6b reveals that the part location within the build chamber also played a critical role in dimensional accuracy. Components fabricated in the inner region exhibited the lowest dimensional deviations, with values as low as 0.05 ± 0.003 mm, due to stable thermal conditions and slower cooling rates within the central region of the powder bed. The middle-region components showed moderate deviation values ranging from 0.09 ± 0.005 mm to 0.13 ± 0.006 mm, while the outer-region components displayed the highest dimensional deviations, reaching 0.17 ± 0.009 mm. The higher deviations observed near the chamber boundaries are mainly associated with rapid heat dissipation and uneven cooling conditions, which promote localized shrinkage and dimensional instability. Also, as shown in Table 3, the ANOVA results indicate that both factors significantly influence DD, as all *p*-values were <0.05.

Furthermore, the relatively low standard deviation values observed across all measurements confirm the repeatability and reliability of the dimensional characterization process. The combined influence of build orientation and part location revealed that components fabricated in the Y-orientation and positioned within the inner build region demonstrated the best dimensional accuracy and surface quality among all experimental conditions.

### 3.3. SEM Analysis of Porosity Based on Part Location

**Figure 7:**
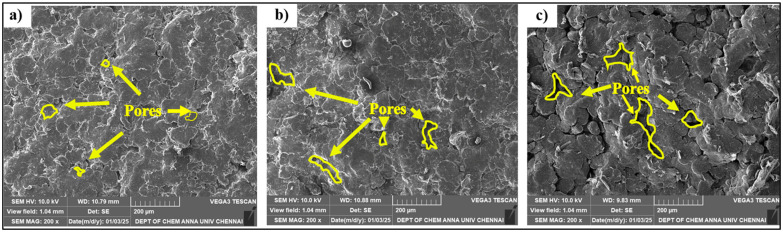
Surface morphology of the components in various part locations: (**a**) inner, (**b**) middle, and (**c**) outer regions.

SEM analysis revealed significant variations in porosity based on part location within the build chamber. Components fabricated in the inner region (Figure 7a) showed fewer pores, smoother fusion boundaries, and minimal surface porosity compared to those in the middle and outer zones. These inner parts exhibited well-sintered surfaces with densely packed spherulitic structures and fewer unmolten particles, indicating efficient heat transfer and stable crystallization. The slower cooling in the inner zones supports the development of uniform lamellar orientations and reduces surface irregularities. Conversely, components built near the outer edges of the chamber (Figure 7c) were subjected to uneven thermal exposure due to their proximity to cooler chamber walls, resulting in rapid cooling. It caused non-uniform crystallization, restricted molecular mobility, and increased surface defects [34]. SEM images of these regions revealed a higher number of voids, incompletely fused particles, and rougher textures. These differences underscore the impact of part location on surface finish and dimensional accuracy in the SLS process. Therefore, SEM observations revealed that specimens fabricated in the outer regions exhibited increased partially fused particles and pore formation compared to samples fabricated in the inner regions. These morphological variations are associated with uneven thermal distribution and rapid cooling behavior within the build chamber, which influenced interlayer fusion and surface quality.

### 3.4. DSC Analysis of Part Location-Dependent Crystallinity

**Figure 8:**
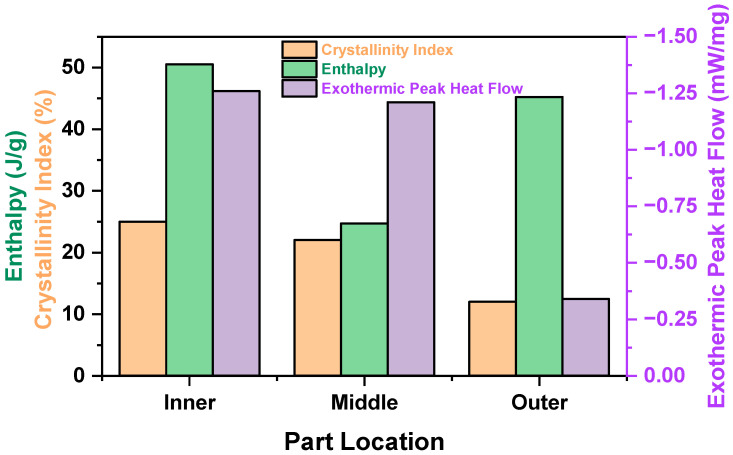
Crystallinity index (%) effect on components at various part locations.

Differential Scanning Calorimetry (DSC) analysis showed significant variations in crystallinity among samples placed at different build chamber locations (Figure 8). The inner-positioned part had the highest Crystallinity Index (CI = 24.16%) as shown in Table 4, indicating a thermally stable zone that supports uniform crystal growth and improved surface quality. Such conditions promote uniform crystal growth and a more ordered molecular structure, resulting in lower surface roughness and improved material consolidation. These results align with previous studies highlighting the benefits of consistent thermal management in SLS processes [35,36]. The middle region exhibited slightly lower crystallinity (CI = 21.63%), likely due to moderate thermal fluctuations and faster edge cooling, resulting in minor inconsistencies. In contrast, the outer-positioned part exhibited the lowest crystallinity (CI = 11.82%), indicating irregular heat exposure, rapid cooling, and disturbed crystallization, which resulted in greater surface roughness.

These findings underline the impact of part placement within the build chamber. Outer regions, being more thermally unstable, promote shrinkage and poor molecular alignment, which affects the surface finish and mechanical integrity [37]. Hence, positioning components in thermally stable areas is critical for improving crystallinity, reducing surface roughness, and enhancing the structural quality of SLS-fabricated polyamide components. Therefore, DSC results indicated slight variations in crystallinity among the fabricated samples depending on build orientation and chamber location. Samples with relatively higher crystallinity exhibited improved fusion characteristics and comparatively lower surface roughness values. In contrast, reduced crystallinity was associated with incomplete fusion behavior and increased surface irregularities.

## 4. Conclusions

The current study confirms that build orientation and part placement have a significant impact on the surface roughness and dimensional Deviation of SLS-fabricated PA12 components. The following observations are listed below:

- Components built in the Y-orientation and located at the inner region of the build platform achieved the best surface finish (S_a_ = 9.88 ± 0.16 µm) with minimal dimensional Deviation (0.05 mm).
- On the other hand, components fabricated in the Z-orientation near the platform edges exhibited the roughest surfaces (S_a_ = 14.08 ± 0.41 µm) and the most considerable dimensional deviations (up to 0.17 mm), attributed to stair-stepping and uneven cooling.
- SEM observations revealed superior microstructural quality and reduced porosity in inner-positioned components. In contrast, outer-located components showed poor fusion and higher porosity.
- DSC results further validated this, showing a crystallinity index of 24.16% for the inner components and only 11.82% for the outer components. These findings provide valuable guidance for optimizing build strategies, enabling SLS manufacturers to enhance the dimensional accuracy and surface quality of the fabricated components.

## Figures and Tables

**Table 1:** Taguchi L9 orthogonal array factors and responses.

Experimental Run	Factor 1	Factor 2	Response 1	Response 2	Response 3	Response 4
	Build Orientation	Part Location	S_a_ (µm)	S_q_ (µm)	S_z_ (µm)	Dimensional Deviation (mm)
1	X	Inner	9.98 ± 0.18	12.22 ± 0.31	65.72 ± 1.84	0.05 ± 0.003
2	X	Middle	10.99 ± 0.24	15.41 ± 0.42	70.84 ± 2.13	0.11 ± 0.006
3	X	Outer	12.07 ± 0.29	16.69 ± 0.51	80.64 ± 2.56	0.15 ± 0.008
4	Y	Inner	9.88 ± 0.16	11.79 ± 0.28	48.92 ± 1.62	0.06 ± 0.004
5	Y	Middle	10.19 ± 0.21	14.83 ± 0.37	61.54 ± 1.95	0.09 ± 0.005
6	Y	Outer	11.49 ± 0.27	15.33 ± 0.45	76.13 ± 2.41	0.14 ± 0.007
7	Z	Inner	12.32 ± 0.33	17.53 ± 0.58	76.95 ± 2.67	0.09 ± 0.005
8	Z	Middle	13.02 ± 0.36	19.04 ± 0.63	102.49 ± 3.14	0.13 ± 0.006
9	Z	Outer	14.08 ± 0.41	20.59 ± 0.71	110.54 ± 3.48	0.17 ± 0.009

**Table 2:** ANOVA for surface roughness parameters.

Surface Parameter	Source	DF	Contribution (%)	Adj SS	Adj MS	F-Value	*p*-Value (<0.05)	Significance
S_a_ (Average)	Build Orientation	2	66.83	11.6432	5.8215	174.22	0.0001	Significant
	Part Location	2	30.71	5.0885	2.5442	76.14	0.001	Significant
	Error	4	0.79	0.1337	0.0337			Not Significant
	Total	8	100					
S_z_ (Max)	Build Orientation	2	63.29	1845.3	922.63	26.49	0.005	Significant
	Part Location	2	31.93	931.0	465.48	13.37	0.017	Significant
	Error	4	4.78	139.3	34.83			Not Significant
	Total	8	100					
S_q_ (RMS)	Build Orientation	2	66.37	44.647	22.323	79.94	0.001	Significant
	Part Location	2	31.97	21.505	10.752	38.50	0.002	Significant
	Error	4	1.66	1.117	0.279			Not Significant
	Total	8	100					

**Table 3:** ANOVA for dimensional Deviation.

Parameter	Source	DF	Contribution (%)	Adj SS	Adj MS	F-Value	*p*-Value (<0.05)	Significance
Dimensional Deviation (DD)	Build Orientation	2	18.28	0.0022	0.0111	14.29	0.015	Significant
	Part Location	2	79.16	0.0962	0.0048	61.86	0.001	Significant
	Error	4	2.56	0.0003	0.0001			Not Significant
	Total	8	100					

**Table 4:** DSC analysis of the PA12 samples at various locations.

Sample No	Peak Temperature (°C)	Exothermic Peak Heat Flow (mW/mg)	Enthalpy (J/g)	Crystallinity Index (%)
Inner	179.01	−1.26	50.5	24.16
Middle	179.31	−1.21	24.7	21.63
Outer	179.69	−0.34	45.2	11.82

## Data Availability

The raw data supporting the conclusions of this article will be made available by the authors on request.
